# Location-Price Equilibria when Traditional Retailers Compete Against an Online Retailer

**DOI:** 10.1007/s11151-021-09814-1

**Published:** 2021-05-04

**Authors:** Stefano Colombo, Zemin Hou

**Affiliations:** 1 Department of Economics and Finance, Università Cattolica del Sacro Cuore, Largo Gemelli 1, 20123 Milan, Italy; 2 Department of Industrial Economics, School of Business Administration, Northeastern University, Shenyang, 110169 China

**Keywords:** Location-price game, E-commerce, Hotelling, L1, D4

## Abstract

We consider a location-then-price game where two traditional retailers compete with a location-irrelevant online retailer. We characterize the existing equilibria, and we show that in any possible equilibrium there is direct competition between the traditional retailers. Furthermore, the traditional retailers locate at neither a maximal nor minimal distance. In equilibrium, the price of the online retailer might be higher or lower than the price of the traditional retailers, depending on the relative competitiveness of the online retailer and the traditional retailers.

## Introduction

Online retailers represent a major challenge for traditional (offline) retailers. One crucial difference between online retailers and traditional retailers is that the former are location-irrelevant, whereas the latter are physically located in a specific place. Several empirical studies have shown how the structure of the traditional retailing industries has been re-shaped since the appearance of online competitors (see, for instance, Brynjolfsson and Smith, 2000; Brown and Goolsbee, 2002; Chevalier and Goolsbee, 2003).^1^ Furthermore, the current COVID-19 crisis has also increased the importance of online shopping, given the need to reduce direct contact among consumers (see, for example, Chang and Meyerhoefer, 2020; Pantano et al., 2020). It is expected that this change of habits will last even when the health crisis has subsided (Sheth, 2020).

The existence of online competitors affects the way that traditional retailers compete against each other. The choice of location is a crucial strategic variable for traditional retailers (Aguirregabiria and Vicentini, 2016; Krider and Putler, 2013). Therefore, it is expected that online competition alters the incentives that underlie the location choices of the traditional retailers. However, the theoretical analysis of the location choices of traditional retailers that compete with online retailers is still scant.

In this paper we analyze location-then-price equilibria in a spatial model *a lá* Hotelling (1929), where two traditional retailers compete against an online retailer. The aim of this work is to highlight and describe which forces drive the location choices of traditional retailers when facing the competition of online retailing. Specifically, we address the following question: Does the existence of an online competitor induce the traditional retailers to agglomerate or separate in the space?

We show that only locational equilibria where there is direct competition between the traditional retailers could emerge in equilibrium. Even if both symmetric and asymmetric equilibria are possible, all of them have the following characteristic: the traditional retailers locate in such a way that their equilibrium distance is neither maximal nor minimal. Therefore, traditional retailers position themselves to compete for the middle of the market and the online retailer competes for the extreme ends of the market (sometimes both ends, sometimes only one end).

This paper is rooted in two strands of literature: First, it is rooted in the so-called “spatial competition” literature that investigates the location choices of firms that compete in a spatial dimension (D’Aspremont et al., 1979; Hotelling, 1929).^2^ Second, the present paper is rooted in the literature that investigates the competition between a location-irrelevant retailer and traditional retailers (Balasubramanian, 1998; Bouckaert, 2000; Nakayama, 2009; Colombo and Matsushima, 2020). However, no paper considers the endogenous location of traditional retailers when they compete against an online retailer. This paper aims to fill this gap.^3^

The remainder of the paper proceeds as follows: In Sect. 2 we introduce the model. In Sect. 3 and 4 we derive the equilibria. In Sect. 5 we discuss welfare. In Sect. 6 we consider the case of a multi-channel retailer. Section. 7 concludes.

## The model

In this section we illustrate the model. We consider a unit mass of consumers who are uniformly distributed over a segment of length one. Let us indicate by $x \in [0, \, 1]$ the location of a consumer along the segment. There are three firms that compete in the market: two traditional (offline) retailers, Firm *A* and Firm *B*; and one online retailer, Firm *I*.^4^ The offline retailers are physically located at some point of the segment. Let us indicate by $x_{A} \in [0, \, 1]$ ($x_{B} \in [0, \, 1]$) the location of Firm *A* (*B*). Without loss of generality, we assume that $x_{A} \le x_{B}$. On the other hand, the online retailer has no location.

Each consumer purchases one unit of the good: either from one of the traditional retailers or from the online retailer. The utility function of a consumer who is located at *x* and buys from Firm $i = A,B$ is:

1 $$ u_{i} = v - p_{i} - t\left| {x_{i} - x} \right| $$

where: *v* is the reservation price, which is assumed to be sufficiently high so that the market is always covered in equilibrium; $p_{i}$ denotes the Firm *i*’s price; and $t\left| {x_{i} - x} \right|$ represents the consumer’s transportation cost when visiting the traditional retailer *i*, with $t > 0$.^5^

On the other hand, the utility function of a consumer who buys from Firm *I* is:

2 $$ u_{I} = v - p_{I} - z $$

where *z* can be positive or negative: $z > 0$ indicates the disutility cost of shopping online (for example, the delay in receiving the products, the inability of consumers to inspect the product beforehand, the difficulty in returning products…), whereas $z < 0$ indicates the gain from shopping online (for example, the possibility to obtain targeted services).^6^ We assume that the cost or the gain of purchasing online is the same for all consumers,^7^ and there are no transportation costs: Firm *I* is location-irrelevant. We assume that fixed costs and marginal costs of both the traditional retailers and the online retailer are constant and normalized to zero.

We consider the following two-stage game: In Stage 1, the traditional retailers choose simultaneously where to locate; in Stage 2, both the traditional retailers and the online retailer choose simultaneously their prices. We solve the game by backward induction.

Before proceeding, it should be noted that, depending on the first-period location choice of the traditional retailers, different market structures are possible. We use the following notation: $x_{AB}$ is the consumer who is indifferent between buying from Firm *A* and Firm *B*; $x_{A}^{L}$ ($x_{A}^{R}$) is the consumer who is indifferent between buying from Firm *A* and Firm *I* and is located at the left (right) of Firm *A*; and $x_{B}^{L}$ ($x_{B}^{R}$) is the consumer who is indifferent between buying from Firm *B* and Firm *I* and is located at the left (right) of Firm *B*. They are obtained by equating $u_{A}$ with $u_{B}$, $u_{A}$ with $u_{I}$, and $u_{B}$ with $u_{I}$, respectively. They are: $x_{AB} = \frac{{p_{B} - p_{A} + t(x_{A} + x_{B} )}}{2t}$; $x_{i}^{L} = \frac{{p_{i} - p_{I} + tx_{i} - z}}{t}$; and $x_{i}^{R} = \frac{{p_{I} - p_{i} + tx_{i} + z}}{t}$.

**Fig. 1:**
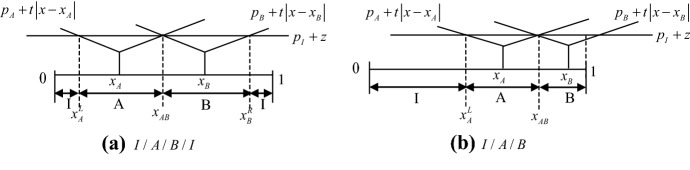
Direct market structures among two traditional retailers and one online retailer

**Fig. 2:**
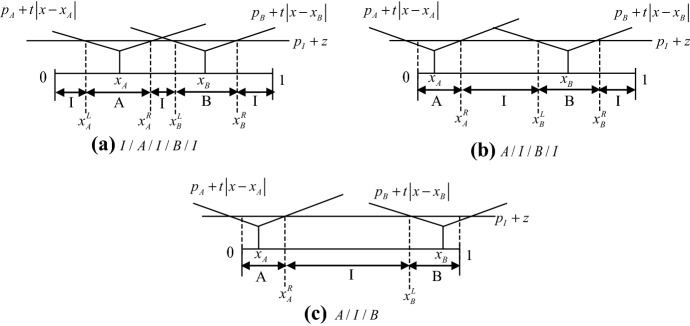
Indirect market structures among two traditional retailers and one online retailer

Depending on the relative location of these indifferent consumers, the following market structures are possible: (1) I/A/B/I, (2) I/A/B, (3) I/A/I/B/I, (4) A/I/B/I, (5) A/I/B (see Figs. 1 and 2).^8^

Intuitively, these five market structures can be classified into two types: Fig. 1 represents the market structures - I/A/B/I and I/A/B - where Firm *A* competes directly with Firm *B*: henceforth, I/A/B/I and I/A/B are indicated as *direct competition* market structures. On the other hand, Fig. 2 represents the market structures - I/A/I/B/I, A/I/B/I, and A/I/B - where Firm *A* does not compete directly with Firm *B*: henceforth, I/A/I/B/I, A/I/B/I, and A/I/B are indicated as *indirect competition* market structures.

The subsequent analysis will show which of these possible market structures can be equilibrium outcomes and their welfare consequences. In particular, in what follows we first consider the *direct competition* market structures, and we show that location-price equilibria exist under this kind of market structure: The configurations in Fig. 1 have stable equilibria. Moreover, we show that when ${z \mathord{\left/ {\vphantom {z t}} \right. \kern-\nulldelimiterspace} t}$ is sufficiently high or low, only the market structure I/A/B emerges, whereas, if ${z \mathord{\left/ {\vphantom {z t}} \right. \kern-\nulldelimiterspace} t}$ is intermediate, both the market structure I/A/B and the market structure I/A/B/I emerge (multiple market structures). Then we consider the *indirect competition* market structures, and we show that no location-price equilibrium exists under this kind of market structure: The market structures in Fig. 2 are unstable.

## Location-Price Equilibria: Direct Competition Market Structures

In this section, we derive the location-price equilibria in the *direct competition* market structures. We first consider case I/A/B/I and then we consider case I/A/B.

### Case I/A/B/I

In case I/A/B/I, the traditional retailer *A* competes directly with the traditional retailer *B* for the interior market, whereas the online retailer *I* serves those consumers who are located close to the boundaries of the market. A consumer who is located at *x* purchases from Firm *r* if $u_{r} > u_{ - r}$ ($r = A,B,I$). Therefore, from Fig. 1a, Firm $r$’s demand – $q_{r}$ – is defined by: $q_{A} = x_{AB} - x_{A}^{L}$; $q_{B} = x_{B}^{R} - x_{AB}$; and $q_{I} = 1 - x_{B}^{R} + x_{A}^{L}$. This yields the following profit function for Firm *r*: $\pi_{r} = p_{r} q_{r}$. In the second period, each firm sets the price in order to maximize its profits, which yields:

3 $$ p_{A} = p_{B} = \frac{{t(1 - x_{A} + x_{B} ) + 2z}}{8};\;\;p_{I} = \frac{{t(5 + 3x_{A} - 3x_{B} ) - 6z}}{16} $$

In the first period, the two traditional retailers *A* and *B* decide where to locate by choosing $x_{A}$ and $x_{B}$. By substituting (3) into the profit functions, it can be observed that $\frac{{\partial \pi_{A} }}{{\partial x_{A} }} = - \frac{{\partial \pi_{B} }}{{\partial x_{B} }} = \frac{{ - 3\left[ {t\left( {1 - x_{A} + x_{B} } \right) + 2z} \right]}}{64} < 0$: The profits of Firm *A* (*B*) are strictly decreasing (increasing) in its location, so that the two traditional retailers locate as far apart as possible given the market structure I/A/B/I, which requires $x_{AB} \le x_{A}^{R}$ and $x_{AB} \ge x_{B}^{L}$. Therefore, by solving $x_{A}^{R} = x_{B}^{L} = x_{AB}$, we get:

4 $$ x_{B,s}^{*} - x_{A,s}^{*} = x_{d,s}^{*} = \frac{3}{13} + \frac{6}{13}\frac{z}{t} $$

where the subscript “*s*” identifies the equilibrium variables in the *symmetric* direct competition market structure I/A/B/I.

The following proposition illustrates the equilibrium location in the market structure I/A/B/I^9^:

#### Proposition 1

Denote $x_{B}^{MIN} \equiv \frac{9t + 18z}{{26t}}$; $x_{B}^{MAX} \equiv \frac{23t - 6z}{{26t}}$; $\underline{x}_{B} \equiv \frac{t - z}{{2t}}$; $\underline{x}_{A} \equiv \frac{t + z}{{2t}}$; $\overline{x}_{B} \equiv \frac{t(130 - 18\sqrt 3 ) + z(13 - 36\sqrt 3 )}{{117t}}$; and $\overline{x}_{A} \equiv \frac{t(18\sqrt 3 - 13) + z(36\sqrt 3 - 13)}{{117t}}$. There are infinite location equilibria that support the market structure I/A/B/I. The range of Firm *B*’s equilibrium location is as follows:

$$ \left\{ {\begin{array}{*{20}l}    {x_{{B,s}}^{ * }  \in \left[ {\underline{x} _{A}  + x_{{d,s}}^{ * } ,\underline{x} _{B} } \right]} \hfill & {if{\text{ }}{z \mathord{\left/ {\vphantom {z t}} \right. \kern-\nulldelimiterspace} t} \in \left[ { - {1 \mathord{\left/ {\vphantom {1 2}} \right. \kern-\nulldelimiterspace} 2}, - 0.21} \right]} \hfill  \\    {x_{{B,s}}^{ * }  \in \left[ {\underline{x} _{A}  + x_{{d,s}}^{ * } ,\underline{x} _{B} } \right]{\text{ }}or{\text{ }}x_{{B,s}}^{ * }  \in \left[ {x_{B}^{{MIN}} ,\overline{x} _{A}  + x_{{d,s}}^{ * } } \right]{\text{ }}or{\text{ }}x_{{B,s}}^{ * }  \in \left[ {\overline{x} _{B} ,x_{B}^{{MAX}} } \right]} \hfill & {if{\text{ }}{z \mathord{\left/ {\vphantom {z t}} \right. \kern-\nulldelimiterspace} t} \in \left[ { - 0.21, - 0.16} \right]} \hfill  \\    {x_{{B,s}}^{ * }  \in \left[ {x_{B}^{{MIN}} ,\overline{x} _{A}  + x_{{d,s}}^{ * } } \right]{\text{ }}or{\text{ }}x_{{B,s}}^{ * }  \in \left[ {\overline{x} _{B} ,x_{B}^{{MAX}} } \right]} \hfill & {if{\text{ }}{z \mathord{\left/ {\vphantom {z t}} \right. \kern-\nulldelimiterspace} t} \in \left[ { - 0.16,0.08} \right]{\text{  }}} \hfill  \\    {x_{{B,s}}^{ * }  \in \left[ {x_{B}^{{MIN}} ,\underline{x} _{B} } \right]{\text{ }}or{\text{ }}x_{{B,s}}^{ * }  \in \left[ {\underline{x} _{A}  + x_{{d,s}}^{ * } ,x_{B}^{{MAX}} } \right]} \hfill & {if{\text{ }}{z \mathord{\left/ {\vphantom {z t}} \right. \kern-\nulldelimiterspace} t} \in \left[ {0.08,0.13} \right]} \hfill  \\    {x_{{B,s}}^{ * }  \in \left[ {\overline{x} _{B} ,\overline{x} _{A}  + x^{*}_{{d,s}} } \right]} \hfill & {if{\text{ }}{z \mathord{\left/ {\vphantom {z t}} \right. \kern-\nulldelimiterspace} t} \in \left[ {0.35,0.45} \right]} \hfill  \\    {x_{{B,s}}^{ * }  \in \left[ {x_{B}^{{MIN}} ,x_{B}^{{MAX}} } \right]} \hfill & {if{\text{ }}{z \mathord{\left/ {\vphantom {z t}} \right. \kern-\nulldelimiterspace} t} \in \left[ {0.45,0.58} \right]} \hfill  \\   \end{array} } \right. $$

whereas Firm *A*’s equilibrium location is $x_{A,s}^{*} = x_{B,s}^{*} - (\frac{3}{13} + \frac{6}{13}\frac{z}{t})$.^10^

#### Proof

See the Appendix.

As is indicated in Proposition 1, for any $x_{B,s}^{*}$, the corresponding Firm *A*’s equilibrium location is $x_{A,s}^{*} = x_{B,s}^{*} - (\frac{3}{13} + \frac{6}{13}\frac{z}{t})$, so that the equilibrium distance between the traditional retailers is constant and equal to $x_{d,s}^{*} = \frac{3}{13} + \frac{6}{13}\frac{z}{t}$. Furthermore, the equilibrium distance coincides with the equilibrium demand of the traditional retailers.^11^ It can be noted that $\frac{{\partial x_{d,s}^{*} }}{\partial t} = - \frac{6z}{{13t^{2} }} < 0$ and $\frac{{\partial x_{d,s}^{*} }}{\partial z} = \frac{6z}{{13t}} > 0$. Indeed, ${z \mathord{\left/ {\vphantom {z t}} \right. \kern-\nulldelimiterspace} t}$ is a measure of the relative competitiveness of the traditional retailers and the online retailer: when ${z \mathord{\left/ {\vphantom {z t}} \right. \kern-\nulldelimiterspace} t}$ is high (low), the traditional retailers are highly (scarcely) competitive with respect to the online retailer. When $z > 0$ and $t$ increases, the equilibrium demand of the traditional retailers decreases, and that of the online retailer increases. At the opposite, when $z < 0$, the online retailer has a huge competitive advantage towards the traditional retailers. As *t* increases, the competitive advantage of the online retailer causes the online retailer to charge higher prices, and this generates a larger demand for the offline retailers.^,^^12^,^13^

Proposition 1 has several implications: When there is an online competitor: (i) There is an infinite set of traditional retailers’ locations that emerge endogenously in equilibrium and support the direct competition market structure where the traditional firms serve the center of the market whereas the online firm serves the boundaries; (ii) the traditional retailers are neither maximally nor minimally differentiated in equilibrium; and (iii) the two traditional retailers need not be symmetric around 1/2.

Proposition 1 shows that the existence of a location-irrelevant online retailer might induce infinite location equilibria. In particular, the existence of an online retailer determines less-than-maximal and more-than-minimal differentiation equilibria, by modifying the standard agglomerating and dispersing forces that drive the locational decision of the traditional retailers.

The intuition is the following: Consider Firm *A*. When deciding whether to move toward Firm *B*, it considers two opposing forces. On the one hand, by moving toward the rival, Firm *A* increases its own demand (*demand effect*); this is an agglomerating force, as it pushes Firm *A* to locate closer to Firm *B*. On the other hand, the traditional retailer *A* does not want to locate too close to Firm *B*, as this magnifies price competition and disrupts its profits. This is the standard *strategic effect*, which pushes the traditional firms away from each other; it is a dispersing force.

Due to the existence of an online retailer, similar forces are also at work when Firm *A* moves toward the endpoint of the segment. In this case, Firm *A* faces the competition of the online retailer. Indeed, when Firm *A* moves toward the left endpoint, it reduces the market share of Firm *I*, which reacts by reducing its price (see (3)). This is similar to the *strategic effect* mentioned above, but now it works as an agglomerating force vis-à-vis Firm B, because it induces Firm *A* to locate toward the center of the market.^14^ At the same time, Firm *A* would like to move toward the left endpoint, in order to steal demand from the online firm; this is similar to the *demand effect* mentioned above, but now it works as a dispersing force vis-a-vis Firm B.^15^

Firm *A* and Firm *B* do not move to the boundaries because the presence of Firm *I* puts a limit on the extent to which the traditional retailers can soften competition and set higher prices by moving away from each other. Furthermore, as the online retailer serves the boundaries of the segment, the traditional retailers (even if identical) need not be located symmetrically around the centre of the market.^16^

The following remark summarizes the above discussion:

#### Remark 1

Consider the location choice of a traditional retailer. The strategic effect and the demand effect in relation to the online retailer are opposed to the strategic effect and the demand effect in relation to the other traditional retailer.

Our result can be compared with Guo and Lai (2017). In Guo and Lai (2017), as a consequence of the entry of an online retailer, the physical retailers move toward more densely populated segments. In our framework, where there is a uniform distribution of consumers along the linear market, the existence of an online retailer determines an intermediate equilibrium distance between the traditional retailers.

Foncel et al. (2011) and Chu et al. (2012) consider the competition between one online retailer and one traditional retailer, and study the location-then-price equilibrium. Similar to our conclusion, these studies find that the traditional retailer tends to locate close to the center of the market whereas the online retailer serves the boundaries.

Now we provide the intuition for the existence of infinite equilibria.^17^ Consider Fig. 1a. For any given *z/t* that satisfies the conditions for I/A/B/I, the distance between $x_{A,s}^{*}$ and $x_{B,s}^{*}$ is specified by $x_{d,s}^{*}$ in (4). Note that there are an infinite number of equal-magnitude minute changes in the traditional firms’ locations (sliding back and forth horizontally along the segment) that yield $x_{d,s}^{*}$ and that also satisfy the condition that Firm *I* sells to consumers who are to the left of $x_{A,s}^{*}$ and to the right of $x_{B,s}^{*}$.

The profits of the retailers at the equilibrium locations are the following:

5 $$ \pi_{A,s}^{*} = \pi_{B,s}^{*} = \frac{{6(t + 2z)^{2} }}{169t},\;\pi^{*}_{I,s} = \frac{{t(5 + 3x_{A} - 3x_{B} ) - 6z}}{16} $$

Therefore, even if there are infinite location equilibria, all of them induce the same profits for both the traditional retailers and the online retailer. Indeed, all of the equilibria are such that the distance between the traditional retailers is the same, and thus yield the same degree of competition between them. For the same reason, the overall demand of the online retailer is identical under any possible equilibrium, and the strength of the competition between any traditional retailer and the online retailer is unaffected by the equilibrium locations.

From (5), the profits of the online retailer strictly increase with *t*, as the competitiveness of Firm *I* increases when *t* goes up all else being equal. In contrast, the profits of the traditional retailers are U-shape in *t*: When *t* increases, there are two contrasting forces: First, an increase in *t* mitigates the competition between the two traditional retailers, which thereby increases their profits; and, second, an increase in *t* diminishes their competitiveness vis-a-vis Firm *I*, which thus reduces their profits. If *t* is high (low), the absolute value of demand variation with respect to *t* is low (high)^18^: The first (second) effect dominates, and their profits increase (decrease) with *t*.

Finally, the profits of the traditional retailers increase with *z*, whereas the profits of the online retailer decrease with *z*. Indeed, when *z* is high (low), the traditional retailers have a relative competitive (dis-)advantage over the online retailer; the traditional retailers thus get greater (smaller) profits, whereas the opposite holds for the online retailer.

The next proposition compares the equilibrium prices of the online retailer and the traditional retailers:

#### Proposition 2

Within the market structure I/A/B/I, the online retailer’s equilibrium price is higher (lower) than the traditional retailers’ equilibrium price when ${z \mathord{\left/ {\vphantom {z t}} \right. \kern-\nulldelimiterspace} t} \le ( \ge ) \, {3 \mathord{\left/ {\vphantom {3 {20}}} \right. \kern-\nulldelimiterspace} {20}}$.

#### Proof

Note that $p_{I,s}^{*} \ge ( \le ) \, p_{A,s}^{*} = p_{B,s}^{*}$ if and only if ${z \mathord{\left/ {\vphantom {z t}} \right. \kern-\nulldelimiterspace} t} \le ( \ge ) \, {3 \mathord{\left/ {\vphantom {3 {20}}} \right. \kern-\nulldelimiterspace} {20}}$.

Therefore, the equilibrium price of the online retailer might be either higher or lower than the equilibrium price of the traditional retailers, depending on *z/t*. When *z* is quite high relative to *t*, the online price is expected to be lower: When the online retailer has a relative competitive disadvantage over the traditional retailers, it is more likely that it sets a lower price to attract consumers, while the opposite is true when it has a relative competitive advantage (for a similar argument, see, for example, Smith et al., 2000). Note that a similar result is also found in Guo and Lai (2017).^19^

### Case I/A/B (Case A/B/I)

In this section we consider case I/A/B (case A/B/I can be treated identically). In case I/A/B we still have direct competition between traditional retailers. However, now only Firm *A* competes against the online retailer *I*. From Fig. 1b, Firm $r$’s demand, $q_{r}$, is defined by: $q_{A} = x_{AB} - x_{A}^{L}$; $q_{B} = 1 - x_{AB}$; and $q_{I} = x_{A}^{L}$. The profit function for Firm *r* is $\pi_{r} = p_{r} q_{r}$. By maximizing the profit functions with respect to prices, we get the following equilibrium prices:

6 $$ p_{A} = \frac{{t(2 - x_{A} + x_{B} ) + 2z}}{9};\;\;p_{B} = \frac{{t(10 - 5x_{A} - 4x_{B} ) + z}}{9};\;\;p_{I} = \frac{{t(2 + 8x_{A} + x_{B} ) - 7z}}{18} $$

By inserting the equilibrium prices into the profit functions and then maximizing with respect to locations, we derive the equilibrium locations in the next proposition, where the subscript “*a*” is used to indicate the equilibrium variables in the *asymmetric* direct competition market structure, I/A/B:

#### Proposition 3

Provided that $- 1 < {z \mathord{\left/ {\vphantom {z t}} \right. \kern-\nulldelimiterspace} t} < {{35} \mathord{\left/ {\vphantom {{35} {44}}} \right. \kern-\nulldelimiterspace} {44}}$, there is a (unique) location equilibrium that supports the market structure I/A/B: $x_{A,a}^{*} = \frac{50t - 29z}{{79t}}$; and $x_{B,a}^{*} = \frac{72t - 7z}{{79t}}$.^20^

#### Proof

See the Appendix.

Proposition 3 shows that when the relative competitive advantage of the online retailer over the traditional retailers is moderately high (i.e. $- 1 < {z \mathord{\left/ {\vphantom {z t}} \right. \kern-\nulldelimiterspace} t} < {{35} \mathord{\left/ {\vphantom {{35} {44}}} \right. \kern-\nulldelimiterspace} {44}}$), it is always possible to identify a (unique) pair of asymmetric traditional retailers’ locations that sustain the market structure I/A/B.

The equilibrium location of Firm *A* is the result of the demand effect and the strategic effect toward both the other traditional retailer and the online retailer. As explained above (see Remark 1), the demand effect is an agglomerating (dispersing) force, and the strategic effect is a dispersing (agglomerating) force when Firm *A* is considering competition against Firm *B* (*I*). On the other hand, Firm *B* faces competition only from Firm *A*, and its equilibrium location is the result of the demand effect and the strategic effect toward the traditional rival.^21^ It should be emphasized that an asymmetric location equilibrium emerges even if the traditional retailers are ex-ante identical.^22^

Finally, note that the necessary condition for the market structure I/A/B to emerge in equilibrium is that the online retailer (traditional retailers) is (are) sufficiently competitive with respect to the traditional retailers (online retailer) (i.e. $- 1 < {z \mathord{\left/ {\vphantom {z t}} \right. \kern-\nulldelimiterspace} t} < {{35} \mathord{\left/ {\vphantom {{35} {44}}} \right. \kern-\nulldelimiterspace} {44}}$). Indeed, if the online retailer (traditional retailers) is (are) scarcely competitive, its (their) demand is zero at the equilibrium prices.

At the locational equilibrium, the profits are:

7 $$ \pi_{A,a}^{*} = \frac{{600(t + z)^{2} }}{6241t},\;\;\pi_{B,a}^{*} = \frac{{392(t + z)^{2} }}{6241t},\;\;\pi_{I,a}^{*} = \frac{{(35t - 44z)^{2} }}{6241t} $$

It can be observed that Firm *A* gets higher profits than does Firm *B*: Firm *B* is constrained at the right, so its equilibrium demand is smaller than that of Firm *A*.^23^ At the same time, the traditional retailer *A* always gets higher profits in the market structure I/A/B than in the market structure I/A/B/I: Firm *A*, by pushing Firm *B*’s market territory to the right endpoint, gets a larger demand and thus greater profits than under I/A/B/I.

On the other hand, when *z*/*t* is sufficiently low, Firm *B* also gets larger profits in the I/A/B structure: Firm *B* faces a trade-off: On the one hand, since it is constrained at the right of the segment, its demand is smaller in the I/A/B structure^24^; on the other hand, it is protected from the direct competition of the online retailer. When the online retailer is highly competitive relative to the traditional retailers − *z*/*t* is low – the latter effect dominates, so the profits of Firm *B* are larger in the I/A/B structure than in the I/A/B/I structure, while the opposite is true when the online retailer is scarcely competitive (that is, *z*/*t* is high).^25^

Note that Firm *B* does not deviate to induce a different market structure when *z*/*t* is high. Although Firm *B* gets smaller profits in I/A/B than in I/A/B/I, a deviation from Firm *B* to induce I/A/B/I is not profitable. Indeed, given the location of Firm *A* at $x_{A,a}^{*}$, when Firm *B* moves to the left to induce I/A/B/I, the resulting distance between the two firms is $\frac{227}{{869}} - \frac{10}{{869}}\frac{z}{t}$, which is lower than the equilibrium distance between the two firms in the I/A/B structure, which is $\frac{22}{{79}} + \frac{22}{{79}}\frac{z}{t}$. Therefore, the demand of Firm *B* is lower after the deviation, thus making the deviation not profitable.^26^, ^27^

The following proposition compares the equilibrium prices of the retailers in the I/A/B market structure:

#### Proposition 4

Within the market structure I/A/B, the online retailer’s equilibrium price is higher (lower) than Firm *A*’s equilibrium price when ${z \mathord{\left/ {\vphantom {z t}} \right. \kern-\nulldelimiterspace} t} \le ( \ge ) \, {{15} \mathord{\left/ {\vphantom {{15} {64}}} \right. \kern-\nulldelimiterspace} {64}}$; and it is higher (lower) than Firm *B*’s equilibrium price when ${z \mathord{\left/ {\vphantom {z t}} \right. \kern-\nulldelimiterspace} t} \le ( \ge ) \, {7 \mathord{\left/ {\vphantom {7 {72}}} \right. \kern-\nulldelimiterspace} {72}}$.

#### Proof

Note that $p_{I,a}^{*} \ge ( \le ) \, p_{A,a}^{*}$ if and only if ${z \mathord{\left/ {\vphantom {z t}} \right. \kern-\nulldelimiterspace} t} \le ( \ge ) \, {{15} \mathord{\left/ {\vphantom {{15} {64}}} \right. \kern-\nulldelimiterspace} {64}}$; and $p_{I,a}^{*} \ge ( \le ) \, p_{B,a}^{*}$ if and only if ${z \mathord{\left/ {\vphantom {z t}} \right. \kern-\nulldelimiterspace} t} \le ( \ge ) \, {7 \mathord{\left/ {\vphantom {7 {72}}} \right. \kern-\nulldelimiterspace} {72}}$.

Indeed, if *z* is high relative to *t*, the relative competitive disadvantage of Firm *I* forces it to set a lower price to attract consumers.

Finally, by jointly considering the parameter conditions that support the market structures I/A/B/I and I/A/B, we can state the following corollary:

#### Corollary of Proposition 1 and 3

When the market structure I/A/B/I emerges in equilibrium, the structure I/A/B also emerges in equilibrium, while the reverse is not necessarily true. In particular, when ${z \mathord{\left/ {\vphantom {z t}} \right. \kern-\nulldelimiterspace} t} \in [ - 1, - {1 \mathord{\left/ {\vphantom {1 2}} \right. \kern-\nulldelimiterspace} 2}] \cup [{7 \mathord{\left/ {\vphantom {7 {12}}} \right. \kern-\nulldelimiterspace} {12}},{{35} \mathord{\left/ {\vphantom {{35} {44}}} \right. \kern-\nulldelimiterspace} {44}}]$, only the market structure I/A/B emerges in equilibrium, whereas when $- {1 \mathord{\left/ {\vphantom {1 2}} \right. \kern-\nulldelimiterspace} 2} \le {z \mathord{\left/ {\vphantom {z t}} \right. \kern-\nulldelimiterspace} t} \le {7 \mathord{\left/ {\vphantom {7 {12}}} \right. \kern-\nulldelimiterspace} {12}}$ both the market structure I/A/B/I and the market structure I/A/B emerge in equilibrium.

#### Proof

A necessary (but not sufficient) condition for the structure I/A/B/I to emerge in equilibrium is that $- 1 < - {1 \mathord{\left/ {\vphantom {1 2}} \right. \kern-\nulldelimiterspace} 2} < {z \mathord{\left/ {\vphantom {z t}} \right. \kern-\nulldelimiterspace} t} < {7 \mathord{\left/ {\vphantom {7 {12}}} \right. \kern-\nulldelimiterspace} {12}} < {{35} \mathord{\left/ {\vphantom {{35} {44}}} \right. \kern-\nulldelimiterspace} {44}}$. It follows that if the necessary and sufficient condition for I/A/B/I to occur is satisfied, then the necessary and sufficient condition for I/A/B to occur – $- 1 < {z \mathord{\left/ {\vphantom {z t}} \right. \kern-\nulldelimiterspace} t} < {{35} \mathord{\left/ {\vphantom {{35} {44}}} \right. \kern-\nulldelimiterspace} {44}}$ – is satisfied as well, while the opposite does not necessarily hold.

The above corollary clarifies that when the *symmetric* direct competition market structure I/A/B/I emerges in equilibrium, then also the *asymmetric* direct competition market structure I/A/B (and A/B/I) emerges in equilibrium, while the reverse is not necessarily true. In other words, I/A/B/I cannot be a unique equilibrium: Both I/A/B/I and I/A/B are equilibrium outcomes when $- {1 \mathord{\left/ {\vphantom {1 2}} \right. \kern-\nulldelimiterspace} 2} < {z \mathord{\left/ {\vphantom {z t}} \right. \kern-\nulldelimiterspace} t} < {7 \mathord{\left/ {\vphantom {7 {12}}} \right. \kern-\nulldelimiterspace} {12}}$.

## Location-Price Equilibria: Indirect Competition Market Structures

In this section, we move to the *indirect competition* market structures illustrated in Fig. 2, in which each traditional retailer competes only with the online retailer. We state the following proposition:

### Proposition 5

No indirect competition market structure is an equilibrium.

### Proof

See the Appendix.

Proposition 5 states that no equilibrium exists where there is no direct competition between the traditional retailers. Indeed, at any possible *indirect competition* market structure, at least one traditional retailer always has the incentive to deviate by changing location in order to induce another market structure.

Consider the structure I/A/I/B/I, where each traditional retailer competes with the online retailer at both sides. When Firm *A* moves to the right in order to induce I/A/B/I, it competes against the online retailer only at the left, which thus reduces the fierceness of competition and yields higher profits.^28^, ^29^

On the other hand, in cases A/I/B/I and A/I/B, Firm *A* faces price competition from the online retailer only at one side. When moving to the right to induce I/A/B/I and I/A/B, respectively, Firm *A* still competes with the online retailer only at one side, but now it also competes with the other traditional retailer. First, it could be noted that by moving to the right (given Firm *B*’s location), Firm *A* expands its demand.^30^ Second, when ${z \mathord{\left/ {\vphantom {z t}} \right. \kern-\nulldelimiterspace} t}$ is low – the online retailer is quite efficient – competing also against Firm *B* reduces the overall competitive pressure on Firm *A*, which can set a higher price and get higher profits. In this case, deviation is certainly profitable. Instead, when ${z \mathord{\left/ {\vphantom {z t}} \right. \kern-\nulldelimiterspace} t}$ is high, competing also against Firm *B* increases the overall competitive pressure on Firm *A*, which sets a lower price. However, the demand increase after deviation outweighs the price reduction, and thus makes deviation profitable for Firm *A*.

## Welfare

Sections 3 and 4 have shown that the *direct competition* market structures – I/A/B/I and I/A/B – are the only two possible market structures in equilibrium. In this section we discuss the welfare implications of online versus offline competition under I/A/B/I and I/A/B. Note that, given the assumption of complete market coverage, welfare is related to total costs, which are composed of transportation costs (when the consumers buy from the traditional retailers) and by disutility costs or convenience gain (when the consumers buy from the online retailer). Therefore, the total costs are^31^:

8 $$ TC = zq_{I}^{*} + \int_{{x_{A}^{L*} }}^{{x_{AB}^{*} }} {t\left| {x - x_{A}^{*} } \right|dx} + \int_{{x_{AB}^{*} }}^{{x_{B}^{R*} }} {t\left| {x - x_{B}^{*} } \right|dx} $$

The first term in (8), which can be positive or negative, is the total disutility cost or convenience gain from shopping online, whereas the second and third terms indicate the total transportation costs.

When *z* denotes the disutility cost, there are two contrasting forces that affect welfare: On the one hand, there is a direct effect, such that when *t* (*z*) goes up, given the same demand of each firm, the transportation costs (the disutility costs) increase. However, when *t* (*z*) increases, the markets shares are reallocated in a way such that the demand of the traditional retailers (online retailer) is reduced. This is the indirect effect, such that when *t* (*z*) goes up, the transportation (disutility) costs decrease.

When *z* denotes the convenience gain, on the one hand, there is a direct effect, such that when *t* (*z*) goes up, given the same demand of each firm, the transportation costs (the convenience gain) increase (decrease). However, when *t* (*z*) increases, the demand of the traditional retailers (online retailer) is increased (reduced) (see the discussion in Sect. 3). This is the indirect effect, such that when *t* (*z*) goes up, the transportation costs (convenience gain) increase (decrease).

In the case of an increase of *t*, the direct effect always dominates, and the total costs strictly increase with *t*, whereas in the case of an increase of *z*, the direct effect dominates the indirect effect when *z* is sufficiently low; this explains the inverse U-shape relationship between the total costs and *z*.

Furthermore, we can state the following proposition:

### Proposition 6

Welfare is higher in I/A/B/I than in I/A/B.

### Proof

Note that $$TC_{I/A/B} - TC_{I/A/B/I} = \frac{{640240z^{2} - 240744tz + 36443t^{2} }}{2109458t} > 0$$.

The total cost depends on the disutility costs (or convenience gain) and the transportation costs. The former depend on the equilibrium demand of the online retailer, whereas the latter depend on the demand of the traditional retailers and their locations. When $z > 0$ and ${z \mathord{\left/ {\vphantom {z t}} \right. \kern-\nulldelimiterspace} t}$ is small, the disutility costs are larger in I/A/B/I, and the transportation costs are larger in I/A/B; when ${z \mathord{\left/ {\vphantom {z t}} \right. \kern-\nulldelimiterspace} t}$ is large, the disutility costs are larger in I/A/B, and the transportation costs are larger in I/A/B/I. When ${z \mathord{\left/ {\vphantom {z t}} \right. \kern-\nulldelimiterspace} t}$ is small, the transportation costs are more important than the disutility costs (as *t* is large relative to *z*), while the opposite is true when ${z \mathord{\left/ {\vphantom {z t}} \right. \kern-\nulldelimiterspace} t}$ is large. It follows that welfare is greater in I/A/B/I than in I/A/B for any ${z \mathord{\left/ {\vphantom {z t}} \right. \kern-\nulldelimiterspace} t}$. When $z < 0$, both the convenience gain and the transportation costs are larger in I/A/B. However, the transportation cost effect dominates: It follows that welfare is greater in I/A/B/I than in I/A/B for any ${z \mathord{\left/ {\vphantom {z t}} \right. \kern-\nulldelimiterspace} t}$.^32^

In what follows, we consider optimal locations – that is the locations of the physical retailers that maximize welfare – and we compare with the equilibrium locations. We focus on the I/A/B/I market structure. By minimizing $TC_{I/A/B/I}$ with respect to the locations of the traditional retailers, we get:

9 $$ x_{A}^{W} = 1 - x_{B}^{W} = \frac{37t - 39z}{{89t}} $$

so that the minimum total costs are: $\min TC_{I/A/B/I} = \frac{{236zt - 312z^{2} + 9t^{2} }}{356t}$. In equilibrium, the maximum level of welfare is obtained at ${z \mathord{\left/ {\vphantom {z t}} \right. \kern-\nulldelimiterspace} t} = {3 \mathord{\left/ {\vphantom {3 {20}}} \right. \kern-\nulldelimiterspace} {20}}$. By using (4) and restricting attention to symmetric locations around 1/2, we have that it must be $x_{A,s}^{*} = 1 - x_{B,s}^{*} = \frac{5t - 3z}{{13t}}$. Note that $x_{A,s}^{*} \ge ( \le ) \, x_{A}^{W}$ when ${z \mathord{\left/ {\vphantom {z t}} \right. \kern-\nulldelimiterspace} t} \ge ( \le ) \, {3 \mathord{\left/ {\vphantom {3 {20}}} \right. \kern-\nulldelimiterspace} {20}}$.

The following proposition summarizes this result:

### Proposition 7

In the market structure I/A/B/I, when *z*/*t* is relatively high (low), the traditional retailers locate too close to each other (too far apart) with respect to the optimum.

We know from Sect. 3 that the prices of all of the firms are the same when ${z \mathord{\left/ {\vphantom {z t}} \right. \kern-\nulldelimiterspace} t} = {3 \mathord{\left/ {\vphantom {3 {20}}} \right. \kern-\nulldelimiterspace} {20}}$. In this case, the equilibrium market shares of the firms depend only on the weight of costs/gains from online purchasing relative to the transportation costs. In contrast, when ${z \mathord{\left/ {\vphantom {z t}} \right. \kern-\nulldelimiterspace} t} > ( < ){3 \mathord{\left/ {\vphantom {3 {20}}} \right. \kern-\nulldelimiterspace} {20}}$, the price of the offline retailers is higher (lower) than that of the online retailers and the offline retailers are too close (far apart from) each other: Since the equilibrium price of the traditional firms is higher (lower), the traditional firms have less (greater) incentive to enlarge the demand: The *demand effect* is lower (higher).

## Extension: Multi-Channel Retailer

In this section we consider the case of a multi-channel retailer. This means that the same firm owns both a traditional retailer and the online retailer (Cattani et al., 2006). In particular, we assume that Firm *B* merges with Firm *I*, thus creating a multi-channel retailer: Firm *E*. The traditional retailer *A* remains separate from *E*.

We consider two different cases: (*i*) the online store’s price of Firm *E* could be different from the traditional store’s price: Firm *E* can price discriminate; and (*ii*) the online store’s price of Firm *E* must be identical to the traditional store’s price: Firm *E* cannot price discriminate. Since the model is now asymmetric, all of the seven market structures mentioned in Sect. 2 have to be considered.

We can state the following proposition:

### Proposition 8

No location-price equilibrium exists.

### Proof

See the Appendix.

In the case of a multi-channel retailer, neither *direct competition* market structures nor *indirect competition* market structures are robust to deviation. Indeed, for any possible market structure, there is always an incentive for either Firm *A* or Firm *E* – or both of them – to deviate in order to induce another market structure that is more profitable.

With regard to the I/A/B/I market structure, Firm *A* wants to deviate by locating to the right of Firm *B*, in such a way as to induce the market structure I/B/A (see the Appendix). By doing so, Firm *A* avoids two-sided competition (from both the online retailer and its jointly owned traditional retailer). In the Appendix it is also shown that another profitable deviation consists of Firm *A* ‘s moving all the way to the left (at point 0). Intuitively, this again allows Firm *A* to limit the extent of competition from the multichannel firm.

Market structure I/A/B is also not robust, as the multi-channel retailer would deviate by relocating the traditional store to induce either I/B/A/I or I/A/B/I. Under both deviations, Firm *E* creates direct contact between the traditional retailer *B* and the online retailer *I*. Since Firm *B* and Firm *I* belong to the same multi-channel retailer, competition between them is softened. This effect is absent in the market structure I/A/B, as Firm *B* and Firm *I* do not have direct contact.^33^ At the same time, as for the case of independent retailers, *indirect competition* market structures are not robust to deviation as well, since the multi-channel retailer could always re-locate the traditional store in such a way as to induce another preferred market structure.

## Conclusions

Online retailing has revolutionized the economic landscape where traditional (offline) retailers operate. Therefore, the locational choices of the traditional retailers have been affected by the appearance of online competitors. In this paper, we tackle this issue by studying a location-then-price game in a context of competition between two traditional retailers and one location-irrelevant online retailer. The aim of this work is to highlight and describe the forces that drive the location choices of traditional retailers when they are facing the competition of online retailing.

We obtain some interesting results. When there is an online competitor, both symmetric and asymmetric spatial equilibria of the traditional retailers might emerge, but all of them are characterized by: *i*) direct competition between the traditional retailers; and *ii*) moderate distance between the traditional retailers. No indirect competition market structure – where the traditional retailers are separated (such that each of them competes only with the online retailer) – is an equilibrium. On the other hand, competition with the online retailer modifies the demand effect and the strategic effect, so that the balance between these effects occurs at an intermediate distance between the traditional retailers.

The intuition behind the above results is as follows: When a traditional retailer is considering the competition from another traditional retailer, the demand effect induces the former to move toward the other traditional retailer, whereas the strategic effect induces the former to move away from the latter. However, when a traditional retailer also faces competition from an online retailer, the two effects are reversed: Now the demand effect pushes the traditional retailer to move away from the other traditional retailer, whereas the incentive to avoid tough competition with the online retailer (the strategic effect) pushes the traditional retailer to move closer to its traditional rival. The balance between these contrasting forces determines the locational equilibria.

The empirical and anecdotal evidence about the impact of online retailing on physical retailers’ positioning is mixed. Guo and Lai (2017, p. 439) argue that “*numerous physical retailers have moved into urban areas, close to highly dense populations”*. This kind of evidence is supported by our equilibria under the I/A/B/I market structure. However, others believe that online competition would induce traditional retailers to locate in suburban areas (Kotkin, 2001; Ushchev et al., 2015; Kickert and Vom Hofe, 2018; Delage et al., 2020). Dixon and Marston (2002) argue that the internet would place conventional stores in the high-streets, and Li (2010) finds that, with the arrival of online retailers, many bookshops in Sydney located on high streets away from shopping centers. These conclusions are in line with our results under the I/A/B market structure.

When considering the equilibrium prices, we find that the price of the online retailer might be higher or lower than the price of the traditional retailers, depending on the relative competitiveness of the traditional retailers and the online retailers (see also Smith et al., 2000). This is consistent with the contrasting evidence of the impact of online competition on the equilibrium prices of traditional retailers: For example, Brynjolfsson and Smith (2000) find that online prices are 9–16% lower than the prices in conventional outlets, whereas Lee (1998) shows that the prices in online auction markets for used cars are higher than the prices in traditional markets.

Our model could be extended for future research: First, it can be used to analyze the case of downstream competition when one or more manufacturers decide whether to distribute their products through traditional or online retailers, and traditional retailers decide where to locate in the space (Matsushima, 2004).

Second, our setup may be extended to allow demand uncertainty: Suppose that the distribution of consumers along the linear market is unknown rather than uniform. Since demand is uncertain, the incentive for traditional retailers to deviate from a certain market structure would change. This might open the possibility for other locational equilibria.

Third, we can extend our model to take into account multidimensional product differentiation, such as product variety. Indeed, traditional retailers usually provide the most popular goods because of limited shelves and inventories, whereas online retailers can provide both popular and obscure goods. In this case, two-dimensional differentiation in space and variety may change the location-price equilibrium.

Fourth, consumers are often heterogeneous with regard to their ability to shop online, due to different innate skills, age, internet availability…, thus implying that parameter *z* is not identical for all consumers (Colombo and Matsushima, 2020). Allowing double heterogeneity of consumers (with regard to both location and disutility costs/gains from the internet) is likely to modify the location-price equilibria.

Our paper also involves several potential limitations: To start, we assumed full market coverage; this is a common assumption in the literature (see, for example, Balasubramanian, 1998) for reason of tractability. In particular, consider I/A/B/I. Since the utility of *all* of the consumers who purchase from the online retailer is the same and in equilibrium it is identical to the utility of the most distant consumer who purchases from a traditional retailer, if *v* is sufficiently low (so that the participation constraint of the consumers that buy from Firm *I* is binding), the online retailer would be driven out from the market. In other words, we would end up with two traditional retailers serving separate markets.

Second, our model considers a linear market *a là* Hotelling (1929). It is well known that when moving from a linear market to a circular market (Salop, 1979; Vickrey, 1964) or a two-dimensional market (Eaton and Lipsey, 1975) results could change. Therefore, our results might be not robust to other market specifications.

Finally, it should be noted that our model mainly applies to offline versus online competition (or, offline versus “catalog” competition as in Balasubramanian, 1998, or offline versus “mail order” competition as in Bouckaert, 2000). However, the online retailer is located “everywhere” along the horizontal line. Therefore, our model could be interpreted also as the limiting case of the competition between two single-store retailers and a multi-store retailer of different quality (Peng and Tabuchi, 2007). For a similar argument, see also Colombo and Matsushima (2020).

## Footnotes

1 See Brynjolfsson and Smith (2000) for an empirical analysis of books and CDs markets, Brown and Goolsbee (2002) for the life insurance industry, and ECB (2013) for the banking industry.

2 See Colombo (2020) for a recent survey of this literature.

3 To the best of our knowledge, the only paper that addresses endogenous location in a context of more than one traditional retailer is Guo and Lai (2017). However, there are several differences with respect to our work: They consider a simultaneous choice of price and location, rather than a location-then-price game. Furthermore, they assume that the traditional retailers are exogenously asymmetric with regard to the production costs, while we assume symmetry. Finally, they assume that the market is uncovered, while we assume market coverage. Foncel et al. (2011) and Chu et al. (2012) consider competition between *one* traditional retailer and one online retailer.

4 As is common in the literature that considers the competition between traditional retailers and location-irrelevant retailers (Balasubramanian, 1998, Bouckaert, 2000, Nakayama, 2009, Colombo and Hou, 2019, Colombo and Matsushima, 2020, Guo and Lai, 2014 and 2017), we consider only one location-irrelevant (online) retailer. Indeed, several online retailers could generate price undercutting and zero profits.

5 Colombo and Hou (2019) show the impossibility to characterize the location-then-price equilibrium in the case of quadratic transportation costs. See also Guo and Lai (2014).

6 See Colombo and Matsushima (2020) for further examples.

7 See Colombo and Matsushima (2020) for the case of heterogeneity of consumers with regard to the cost or the gain of purchasing online.

8 More precisely, there are seven possible market structures: I/A/I/B/I; I/A/B/I; A/I/B; I/A/B; A/I/B/I; A/B/I; and I/A/I/B. However, as the two traditional retailers are identical, I/A/B and A/B/I, as well as A/I/B/I and I/A/I/B, can be treated symmetrically. Therefore, we can focus only on the five possible market structures that are illustrated in Figs. 1 and 2.

9 The equilibrium price and quantity outcomes are: $p_{A,s}^{*} = p_{B,s}^{*} = \frac{2(t + 2z)}{{13}}$; $p_{I,s}^{*} = \frac{7t - 12z}{{26}}$; $q_{A,s}^{*} = q_{B,s}^{*} = \frac{3(t + 2z)}{{13t}}$; and $q_{I,s}^{*} = \frac{7t - 12z}{{13t}}$. Interestingly, the equilibrium price of the traditional retailers is $\frac{2t}{3}x_{d,s}^{*}$. This relationship between the equilibrium price and the equilibrium distance between the two firms resembles the one in the standard Hotelling game. Suppose that in the standard Hotelling framework, two firms are located at the endpoints of a segment of length *w*, so that the distance between the two firms is *w*. In this case, the equilibrium price is *tw*. Note that in the present model the equilibrium price is just 2*t*/3 of the distance between the firms, because the existence of the online retailer makes competition tougher.

10 The complete expression of the relevant thresholds is reported in the Appendix. The Appendix is available at https://sites.google.com/view/stefanocolombo.

11 Indeed, each firm is located in the middle of its own market and serves the same number of consumers on the left and on the right: $x_{A,s}^{*} = {{(x_{AB,s}^{*} + x_{A,s}^{L*} )} \mathord{\left/ {\vphantom {{(x_{AB,s}^{*} + x_{A,s}^{L*} )} 2}} \right. \kern-\nulldelimiterspace} 2}$ and $x_{B,s}^{*} = {{(x_{B,s}^{R*} + x_{AB,s}^{*} )} \mathord{\left/ {\vphantom {{(x_{B,s}^{R*} + x_{AB,s}^{*} )} 2}} \right. \kern-\nulldelimiterspace} 2}$. It follows that $x_{d,s}^{*} = {{(x_{B,s}^{R*} - x_{A,s}^{L*} )} \mathord{\left/ {\vphantom {{(x_{B,s}^{R*} - x_{A,s}^{L*} )} 2}} \right. \kern-\nulldelimiterspace} 2} = x_{B,s}^{*} - x_{A,s}^{*}$.

12 In particular, when *z/t* > 7/12 the demand of the online store is zero. As *z* > *0* is the disutility cost of purchasing online and *t* is the unit transportation cost, *z/t* < 7/12 requires that the online retailer is sufficiently more competitive than the traditional retailers to have a positive demand in equilibrium. At the opposite, when *z/t* < -0.5 the demand of the offline stores is zero. As *z* < *0* is the additional gain of purchasing online, *z/t* > -0.5 is the necessary condition for the traditional retailers to have a positive demand in equilibrium.

13 $$\frac{{\partial p_{I,s}^{ * } }}{\partial t} = \frac{7}{26} > \frac{2}{13} = \frac{{\partial p_{A,s}^{ * } }}{\partial t} = \frac{{\partial p_{B,s}^{ * } }}{\partial t}$$.

14 Note that “agglomeration” and “dispersion” can only be referred to traditional retailers, as the online firm is location-irrelevant.

15 The result in Proposition 1 could be compared with a recent finding in Pazgal et al. (2016). They also consider a location-then-price game in a Hotelling segment with two traditional retailers, but they allow for an uncovered market. They find that location equilibria with moderate dispersion are possible when the market is uncovered. However, they do not consider the implications of an online retailer.

16 Under the market structure I/A/B/I, firms are symmetric around $\frac{{x_{B,s}^{R} * + x_{A,s}^{L} *}}{2}$ rather than around 1/2.

17 We are in debt with the Editor Larry White for this explanation.

18 In particular, $$\left| {\frac{{\partial (x_{AB,s}^{*} - x_{A,s}^{L*} )}}{\partial t}} \right| = \frac{6z}{{13t^{2} }}$$.

19 Note that the equilibrium price of the online retailer strictly decreases with *z*. Indeed, when *z* is positive, it represents a disadvantage for the consumers when they buy from the online retailer. Therefore, for Firm *I* to serve consumers with a greater *z*, the price of the online retailer must be lower. In contrast, the price of the traditional retailers increases with *z*. The price of the online retailer and the price of the offline retailers equate at ${z \mathord{\left/ {\vphantom {z t}} \right. \kern-\nulldelimiterspace} t} = \, {3 \mathord{\left/ {\vphantom {3 {20}}} \right. \kern-\nulldelimiterspace} {20}}$.

20 The equilibrium outcomes are: $p_{A,a}^{*} = \frac{20(t + z)}{{79}}$; $p_{B,a}^{*} = \frac{28(t + z)}{{79}}$; $p_{I,a}^{*} = \frac{35t - 44z}{{79}}$; $q_{A,a}^{*} = \frac{30(t + z)}{{79t}}$; $q_{B,a}^{*} = \frac{14(t + z)}{{79t}}$; and $q_{I,a}^{*} = \frac{35t - 44z}{{79t}}$.

21 However, it should be noted that Firm *B* faces also the potential competition from the online firm. Therefore, Firm *B* cannot locate too close to Firm *A* under the I/A/B structure; otherwise Firm *I* would serve some consumers who are located close to the right endpoint. In this sense, the mere existence of the online retailer indirectly reduces the strength of the agglomerating incentive of Firm *B* under the I/A/B structure. Furthermore, note that when $z = 0$, the equilibrium distance between the two firms is 22/79, which is greater than 3/13 in the market structure I/A/B/I. Indeed, the two market structures describe different situations: under I/A/B, Firm *B* faces only the competition of the other offline retailer, whereas under I/A/B/I, Firm *B* faces the competition of both the other offline retailer and the online retailer. The absence of competition between the online retailer and Firm *B* in I/A/B reduces the agglomeration incentive of the traditional retailers.

22 Other examples of asymmetric equilibria with ex-ante symmetric firms in the context of spatial competition are Tabuchi and Thisse (1995), Anderson et al. (1997), Braid (1999), Correia-da-Silva and Pinho (2011), Colombo (2016), and Choe et al. (2018).

23 At the same time, the equilibrium price of Firm *B* – $\frac{28(z + t)}{{79}}$ – is higher than the equilibrium price of Firm *A*: $\frac{20(z + t)}{{79}}$. Firm *A* serves more distant consumers, so it sets a lower price than Firm *B*. However, due to larger demand, the profits of Firm *A* are greater than the profits of Firm *B*.

24 The equilibrium demand of Firm *B* is $\frac{3}{13} + \frac{6}{13}\frac{z}{t}$ in the I/A/B/I structure, whereas it is $\frac{14}{{79}}(1 + \frac{z}{t})$ in the I/A/B structure.

25 Interestingly, this implies that there exists a parameter space where both the traditional firms get larger profits in I/A/B/I, thus creating the possibility for the traditional retailers to jointly induce such a market structure. The sustainability of an agreement of this type is left for future research.

26 The equilibrium distance in I/A/B/I, which is equal to $\frac{3}{13} + \frac{6}{13}\frac{z}{t}$, cannot be obtained under deviation, since Firm *A* is fixed at $x_{A,a}^{*}$.

27 A similar argument shows also that a “leapfrogging” deviation by Firm *B* to induce I/B/A/I is not profitable. In addition, deviations that induce *indirect competition* market structures are never profitable (see the Appendix).

28 The price of Firm *A* in I/A/I/B/I is $\frac{t + 4z}{{12}}$, whereas it is $\frac{{2\left( {t + 2z} \right)}}{13}$ in I/A/B/I, with $\frac{t + 4z}{{12}} < \frac{{2\left( {t + 2z} \right)}}{13}$ when ${{ - 1} \mathord{\left/ {\vphantom {{ - 1} 4}} \right. \kern-\nulldelimiterspace} 4} < {z \mathord{\left/ {\vphantom {z t}} \right. \kern-\nulldelimiterspace} t} < {1 \mathord{\left/ {\vphantom {1 2}} \right. \kern-\nulldelimiterspace} 2}$, which are the relevant constraints for the I/A/I/B/I market structure (see the Appendix).

29 A price equilibrium under an *indirect competition* market structure is shown to exist (and to be unique) in the case of maximal differentiation of the traditional retailers in Colombo and Matsushima (2020).

30 The details are in the Appendix.

31 In particular, it is $x_{B}^{R*} = 1$ under I/A/B, whereas there are infinite locational equilibria in I/A/B/I, which, however, yield the same total costs. Furthermore, we have: $TC_{I/A/B/I} = \frac{{9t^{2} + 218tz - 276z^{2} }}{338t}$ and $TC_{I/A/B} = \frac{{274t^{2} + 3313tz - 3202z^{2} }}{6241t}$.

32 In general, it is not easy to identify to whom the increase in welfare when moving from I/A/B to I/A/B/I accrues. Indeed, as shown above, it might be that the profits of one traditional retailer are higher in I/A/B whereas the profits of the other traditional retailer are higher in I/A/B/I, thus making difficult to disentangle which gains from I/A/B/I market structure.

33 It could be observed that under some parameters, Firm *A* also would like to deviate to induce I/A/I/B.
